# Motion area V5/MT+ response to global motion in the absence of V1 resembles early visual cortex

**DOI:** 10.1093/brain/awu328

**Published:** 2014-11-29

**Authors:** Sara Ajina, Christopher Kennard, Geraint Rees, Holly Bridge

**Affiliations:** 1 FMRIB Centre, University of Oxford, UK; 2 Nuffield Department of Clinical Neurosciences, University of Oxford, UK; 3 Wellcome Trust Centre for Neuroimaging, University College London, UK; 4 Institute of Cognitive Neuroscience, University College London, UK

**Keywords:** hemianopia, visual cortex, motion coherence, subcortical, functional MRI

## Abstract

Motion area V5/MT+ shows a variety of characteristic visual responses, often linked to perception, which are heavily influenced by its rich connectivity with the primary visual cortex (V1). This human motion area also receives a number of inputs from other visual regions, including direct subcortical connections and callosal connections with the contralateral hemisphere. Little is currently known about such alternative inputs to V5/MT+ and how they may drive and influence its activity. Using functional magnetic resonance imaging, the response of human V5/MT+ to increasing the proportion of coherent motion was measured in seven patients with unilateral V1 damage acquired during adulthood, and a group of healthy age-matched controls. When V1 was damaged, the typical V5/MT+ response to increasing coherence was lost. Rather, V5/MT+ in patients showed a negative trend with coherence that was similar to coherence-related activity in V1 of healthy control subjects. This shift to a response-pattern more typical of early visual cortex suggests that in the absence of V1, V5/MT+ activity may be shaped by similar direct subcortical input. This is likely to reflect intact residual pathways rather than a change in connectivity, and has important implications for blindsight function. It also confirms predictions that V1 is critically involved in normal V5/MT+ global motion processing, consistent with a convergent model of V1 input to V5/MT+. Historically, most attempts to model cortical visual responses do not consider the contribution of direct subcortical inputs that may bypass striate cortex, such as input to V5/MT+. We have shown that the signal change driven by these non-striate pathways can be measured, and suggest that models of the intact visual system may benefit from considering their contribution.

## Abstract (precis)

Little is known about how non-V1 inputs influence motion area V5/MT+. Ajina *et al.* reveal that after V1 damage, V5/MT+ activity resembles that of early visual cortex, perhaps driven by similar subcortical inputs. While these inputs are normally overshadowed by V1 connections, acknowledging their contribution may improve neuronal models.

## Introduction

A major unsolved problem in our understanding of the human cortex is uncovering the influence of weaker pathways, which may be overshadowed by activity derived from more dominant inputs. By completely removing the influence of these driving cortical areas, the remaining innervations and responses can be studied. In the visual system, the primary visual cortex (V1) has neurons with small receptive fields that are modulated by multiple stimulus characteristics. Areas higher in the visual hierarchy tend to have larger receptive fields, but show increasing stimulus preference. This finding is supported by human studies using population receptive field mapping (Dumoulin and Wandell, 2008; Amano *et al.*, 2009), and is consistent with a feed-forward model of converging V1 signals (Simoncelli and Heeger, 1998; Rust *et al.*, 2006; Wang *et al.*, 2012). However, it is likely that V5/MT+ also possesses direct connections to other cortical and subcortical brain regions. How these alternate inputs are organized and influence V5/MT+ responses remains unknown, yet are essential to understand when developing complete models of the human visual system. Here we address this question by investigating the response of human motion area V5/MT+ following unilateral V1 damage.

In the intact human visual system, blood oxygen level-dependent (BOLD) signals in motion area V5/MT+ increase with increasing global coherence (Rees *et al.*, 2000; Braddick *et al.*, 2001). This is consistent with neurophysiological reports in non-human primates, which show not only that the middle temporal area (MT) is essential for global motion perception (Newsome and Pare, 1988), but that neuronal responses in macaque MT and medial superior temporal area are approximately linearly proportional to coherence (Britten *et al.*, 1993; Heuer and Britten, 2007). In fact, findings in the opposite direction have also been reported in human studies (McKeefry *et al.*, 1997; Smith *et al.*, 2006; Harrison *et al.*, 2007), making an underlying mechanism more difficult to discern and still somewhat unresolved. Despite helpful computational models in the macaque (Rust *et al.*, 2006), attempts to model changes in the BOLD responses remain challenging (Hallum *et al.*, 2011; Kay *et al.*, 2013). Early visual cortical areas, particularly V1, tend to exhibit a negative response to increasing coherence of moving dots, although this has not been as clearly demonstrated as for V5/MT+ and studies have also generated mixed results (Rees *et al.*, 2000; Handel *et al.*, 2007; Costagli *et al.*, 2014).

One possible explanation for the differing responses in these two regions, and in different imaging studies, is the organization of V1 input onto V5/MT+ neurons, which may result in summation of coherent, and suppression of non-coherent motion signals. Where this is not possible or insufficient due to an extremely sparse display, the V5/MT+ response would not get boosted with increasing coherence and may resemble responses in early visual cortex (Harrison *et al.*, 2007).

The influence of both striate and non-striate inputs on V5/MT+ responses can be assessed by comparing neural activity in the visual cortex of patients where V1 has been unilaterally destroyed, with healthy controls where both hemispheres are intact. When V1 is damaged, the response to motion coherence may be determined by interhemispheric interaction or by direct subcortical input to V5/MT+. Extensive research shows that certain aspects of visual information may still undergo processing and influence behaviour even without subjective awareness (Weiskrantz *et al.*, 1974; Leopold, 2012). A number of potential pathways to extrastriate cortex have been postulated, including direct subcortical connections via pulvinar or lateral geniculate nucleus (Sincich *et al.*, 2004; Schmid *et al.*, 2010), as well as callosal connections with the contralateral hemisphere (Bridge *et al.*, 2008).

Here we used functional MRI to investigate the effect of changing motion coherence on human V5/MT+ responses in patients with unilateral damage to V1. By comparing responses in patients elicited by stimulation of the blindfield to both those found in the sighted field, and with healthy control participants, we sought to uncover the influence of inputs to V5/MT+ that are not normally detectable, and to confirm the role of early visual cortex in normal V5/MT+ responses. We predicted that unilateral V1 damage would lead to abolition of the increasing response with coherence in V5/MT+ of the damaged hemisphere. Instead, a dominant direct subcortical input to V5/MT+ should cause coherence-related responses that resemble patterns in V1.

## Materials and methods

### Participants

**Figure 1:**
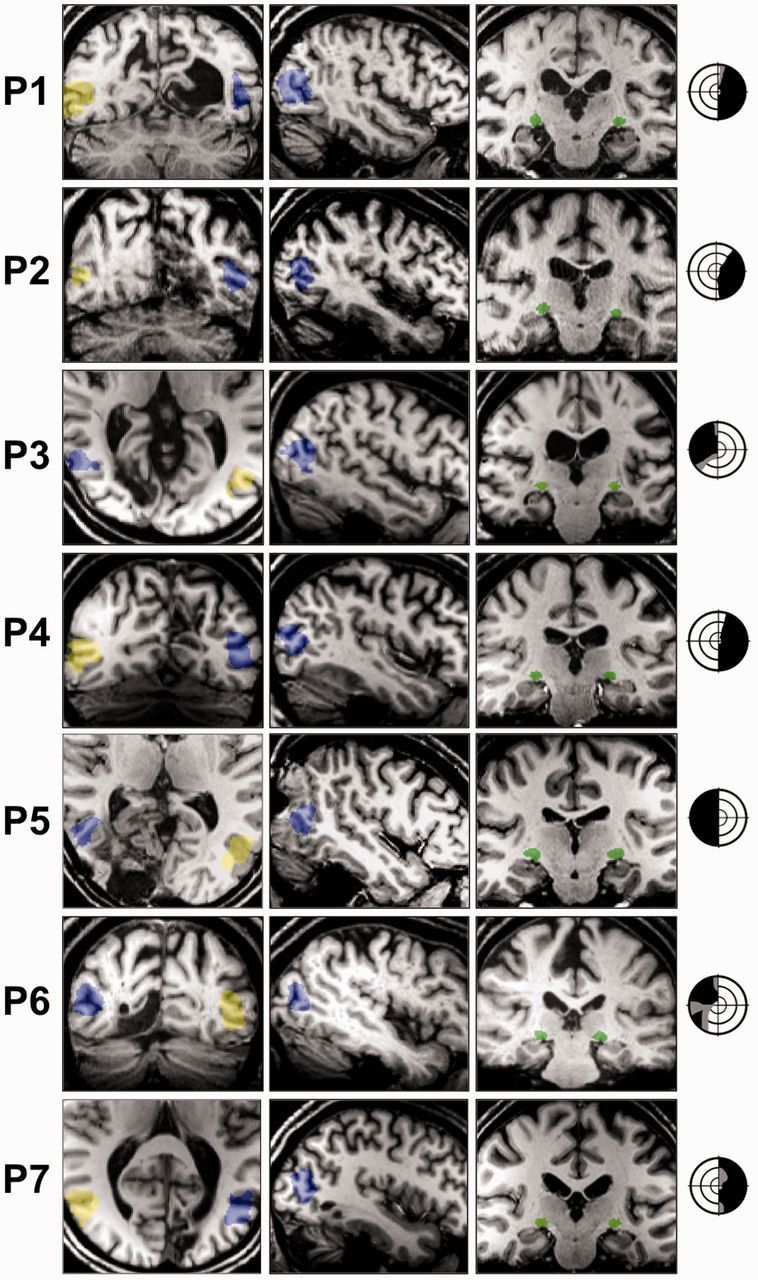
**Patient neuroimaging and visual field deficits**. V5/MT+ masks are overlaid on example coronal or axial (*left* column) and sagittal (*middle* column) T_1_-weighted slices for all seven patients (P1–P7) (radiological convention). Ipsilesional V5/MT+ is shown in blue, contralesional in yellow. Coronal slices (*right* column) also demonstrate the intact lateral geniculate nucleus in green. The lateral geniculate nucleus was identifiable by manual inspection of the anatomical T_1_-weighted images (Horton *et al.*, 1990), with a radiological brain atlas to aid identification. Visual field deficits are adapted from 30:2 threshold Humphrey visual field perimetry reports, and show dense visual field loss in black (<0.5%) and partial loss in grey (<2%). Stimulus location was always restricted to a region of dense visual field loss. Concentric rings represent increments in retinal position of 10°, spanning the central 30°.

Seven individuals were recruited with chronic unilateral damage to V1, causing homonymous visual field loss recorded by Humphrey perimetry (ages 38–76), see Fig. 1 for radiology and visual field schemata in all patients. Pathology was caused by posterior circulation stroke (*n* = 5), or benign tumour resection (*n* = 2), at least 6 months previously (range: 6–252 months post-lesion). Cases with additional non-correctable visual impairment, or prior neurological disease were excluded from the study. Six age-matched participants (ages 25–68) were recruited as controls. All control participants had normal or corrected-to-normal vision, and no history of neurological disease. Participants were recruited from ophthalmological or stroke services in three UK centres. All testing was carried out at the John Radcliffe Hospital, Oxford. Written informed consent was taken, with ethical approval from the Oxford Research Ethics Committee: Ref B 08/H0605/156.

#### Lesion details

Lesion volumes, shown in Table 1, were estimated by creating lesion masks from patients’ T_1_-weighted structural scans. The distribution and extent of damage was also estimated by transforming lobar and subcortical masks from the MNI and Juelich structural atlases to individual structural space using non-linear transformation. We then measured any region of overlap between the lesion and lobe masks, and quantified this as a percentage of the total lobe volume. The subcortical mask incorporated the thalamus (including lateral geniculate nucleus and pulvinar), striatum, and superior colliculi, with an approximate unilateral volume of 50 000 mm^3^. No patients showed any notable involvement of these structures in their lesions (Table 1).

**Table 1:** Patient lesion size and extent

Patient	Total lesion volume (mm^3^)	Occipital damage (%)	Subcortical damage (%)
P1	30 066	38.48	0.00
P2	20 224	15.56	0.00
P3	7080	8.23	0.01
P4	8752	9.22	0.00
P5	28 736	33.33	0.00
P6	15 000	14.63	0.00
P7	16 432	15.79	0.10

No cases showed additional damage to the frontal, parietal, or temporal lobes. Patient P7 did show evidence of additional left cerebellar hemisphere involvement, visible in Fig. 1.

The majority of patients with lesions affecting <20% of the occipital lobe had some small area of V1 sparing. This usually corresponded to the occipital pole, or a region of cortex far anteriorly. These regions would in turn be associated with visual field preservation at the very centre of vision, so called ‘macular sparing’, or in the far periphery. All patients had stimuli presented to their dense region of field loss, which would not be supported by the regions of V1 sparing that were observed radiologically.

No patients showed specific damage to either the lateral geniculate nucleus, or to extrastriate region V5/MT+ in the damaged hemisphere. Figure 1 identifies these regions in all patients, and also suggests that the underlying white matter supporting V5/MT+ should be largely intact.

### Stimuli

**Figure 2:**
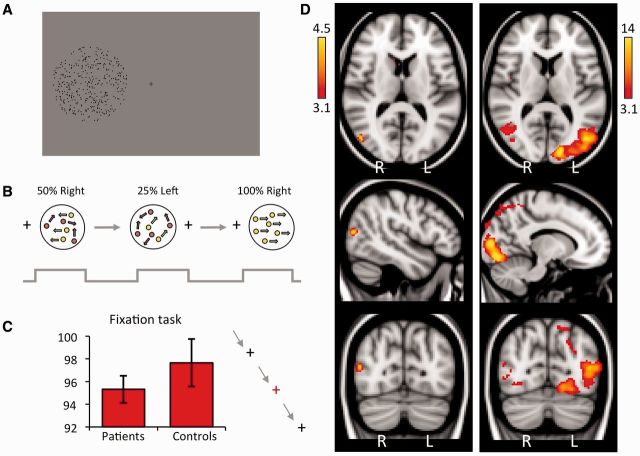
**Experimental design, and average cortical response to visual motion in patients.** (**A**) Experimental stimulus. Participants viewed an aperture containing moving black dots at 8 dots/°^2^ on a grey background, at least 2.5° from fixation. (**B**) The stimulus was presented to each hemifield separately at six coherence levels, with a random sequence 12-block design: 0%, 12.5%, 25%, 50%, 75%, 100%. (**C**) During stimulus presentation, participants were asked to perform a simple task to detect colour changes at fixation. Median performance is shown here for patient and control groups, error bars represent standard error of the mean. (**D**) Thresholded activation maps across all coherence levels, comparing the blind and sighted hemifields of patients. *Left* column: Blind ‘left’ hemifield stimulation. Peak *z*-statistic = 4.46, MNI coordinates: (48, −74, 14). *Right* column: Patients sighted ‘right’ hemifield. Peak *z*-statistic = 18.4, MNI coordinates: (−14, −90, 6). Fixed effects analysis, *P* < 0.001 uncorrected in V1 and extrastriate cortex (cluster extent threshold >10 voxels), elsewhere cluster correction threshold *P* < 0.01, results displayed on MNI standard brain.

Visual stimuli were presented on a 1280 × 1040 resolution monitor at the back of the MRI scanner bore. Participants viewed stimuli via a double mirror mounted on the head coil. When the participant was in position, the screen subtended a visual angle of 23 × 13°. Stimuli were generated using MATLAB (Mathworks) and functions from the Psychophysics Toolbox (Brainard, 1997; Kleiner *et al.*, 2007). Stimuli consisted of an aperture of 5° or 8° diameter, displayed on a uniform grey background. Apertures contained moving black dots at an average density of 8 dots/°^2^, moving at 5°/s. Each dot was 0.075° in diameter, and had a lifetime of 12 frames so it was not possible to infer global direction by tracking individual dots (Fig. 2A). Stimulus location was restricted to the scotoma in patients, a minimum of 2.5° from fixation.

In age-matched controls, we also matched the stimulus size and position to our patient group. Individual cases were paired to controls, with stimulus size and position replicated. In three patients, identical stimulus positions were used (P1, P2 and P5: 8°-diameter aperture, 3° from fixation, on the horizontal meridian), replicated in two controls.

Coherence levels were set at 0%, 12.5%, 25%, 50%, 75% and 100% for each hemifield, representing a 12-condition block design (Fig. 2B). In each block the aperture appeared for 16 s, during which time global motion direction was changed every 2 s (eight directions: 45°, 90°, 135°, 180°, 225°, 270°, 315°, 360°). A 10 s rest period followed each block. There were four runs in total, each lasting 312 s. Throughout the experiment (during condition and rest blocks), participants performed a task to maintain fixation by pressing a button every time the fixation cross changed colour from black to red. Colour changes occurred at random lasting 300 ms, and participants were instructed at the start of the experiment to try not to miss any red crosses. It was also emphasized that they must try to maintain fixation throughout the experiment, and not move their eyes around the screen. An identical procedure was carried out in six age-matched controls and both groups performed at >90% correct in the fixation task (Fig. 2C). An EyeLink 1000 eye tracker (SR Research Limited) was used to confirm fixation by recording eye movements.

### MRI acquisition

Scanning took place in a 3 T Siemens Verio MRI scanner at the Functional Magnetic Resonance Imaging Centre of the Brain (FMRIB, University of Oxford), using a 32-channel head coil. Six hundred and thirty functional volumes were acquired in one single session, duration 21 min (T_2_*-weighted echo-planar imaging, 34 sequential 3 mm slices, repetition time = 2000 ms, echo time = 30 ms, field of view = 192 mm). Magnetization was allowed to reach a steady state by discarding the first five volumes, an automated feature of the scanner. A high-resolution (1 mm× 1 mm× 1 mm voxels) whole-head T_1_-weighted anatomical image (echo time = 4.68 ms, repetition time = 2040 ms, field of view = 200 mm, flip angle = 8°) and a field map with dual echo-time images (echo time 1 = 5.19 ms, echo time 2 = 7.65 ms, whole brain coverage, voxel size 2 mm× 2 mm× 2 mm) was also acquired for each participant.

### Data analysis

Preprocessing and statistical analyses were carried out using tools from FSL (FMRIB’s Software Library, www.fmrib.ox.ac.uk/fsl). Non-brain tissue was excluded from analysis using the Brain Extraction Tool (BET; Smith, 2002), motion correction was carried out using MCFLIRT (Jenkinson *et al.*, 2002), images were corrected for distortion using field maps, spatial smoothing used a Gaussian kernel of 5 mm full-width half-height, and high-pass temporal filtering (Gaussian-weighted least-squares straight line fitting, with sigma = 13.0 s). Functional images were registered to high-resolution structural scans using FLIRT (Jenkinson and Smith, 2001), and a standard MNI brain template using FLIRT and FNIRT (Andersson *et al.*, 2007).

For group analyses it was necessary to align patient brains to a uniform pathological template. Consequently, patients with lesions in the left occipital lobe (*n* = 4) had their structural and functional images flipped in the horizontal plane for subsequent stages of analysis. This meant that as a group (*n* = 7), all patients had sustained damage to the same ‘right’ hemisphere, corresponding to a visual field deficit on the left side of space. Functional images were registered to high resolution scans and the MNI brain template, as described above. All activation coordinates are reported in MNI space, and region identification was determined using the FSLview atlas. It is worth noting that this procedure was useful for displaying group results on canonical brain images, and for model fitting. All graphs, signal change calculations, and correlation statistics were calculated using data from participants’ native space.

### Regions of interest

V5/MT+ masks were derived from anatomically-defined probabilistic maps (Juelich atlas implemented in FSL), non-linearly transformed into functional space for both patients and controls to ensure consistency between participant groups (Fig. 1). In native space, average right region of interest (volume 107 ± 8.5 voxels in patients, 111 ± 11.3 voxels in controls.) Left V5/MT+ was 102 ± 11.3 voxels in patients, 106 ± 18.9 voxels in controls. V1 masks in controls, and in the intact hemisphere of patients, were functionally defined for each participant so that they corresponded to retinotopically-active regions of calcarine cortex. Average V1 region of interest volume was 61 ± 62.2 voxels in patients (undamaged hemisphere), and 108 ± 73.9 voxels in controls.

To measure the effect of motion coherence on BOLD activation, a data-driven model was thought to be most sensitive to any differences between patients and controls. Each of the 12 conditions (left or right hemifield, six coherence levels) were entered into the general linear model as separate explanatory variables and contrasts. We then extracted the percentage change for the contrast of parameter estimate versus baseline within regions of interest at each coherence level for each participant. In controls, this was averaged first across hemispheres, and then across participants to provide a single measure of contralateral and ipsilateral activity in each region. In patients, average signal change was calculated across participants for each hemisphere separately. An additional analysis was performed to measure average BOLD response across all coherence levels for each hemifield separately.

Pearson correlation analyses were used to compare patterns of activation with coherence across regions of interest, and between patients and controls. When comparing activity in the same participants, paired correlation analyses were performed. All tests were two-tailed unless otherwise stated.

### Model fitting

For model fitting, signal change in contralateral V5/MT+ and V1 regions of interest in controls were averaged and normalized, such that the sum of all points was equal to zero. Integers representing points along these curves were then entered as weights into a higher-level general linear model, along with contrast images for all participants. Fixed-effects analyses were carried out for each hemifield separately, in control and patient groups. In the patient group, this analysis was repeated using a mixed effects model to determine whether individual outliers may have skewed the results. A statistical threshold of *P* < 0.001 uncorrected (cluster extent threshold >10 voxels) was used to test for significance within V1 and extrastriate cortex, for which we had *a priori* hypotheses. Elsewhere correction for multiple comparisons was made using a cluster threshold of *P* < 0.01. For this purpose, anatomically-defined probabilistic maps (Juelich atlas implemented in FSL) were used to define these regions of interest (bilateral V1 and extrastriate cortex), total volume = 15 800 mm^3^.

### Eye movements

Eye movements can be a legitimate concern when considering results for visual stimulation inside a scotoma. Three lines of evidence suggest that this was not a problem in our experiment, and could not have accounted for our results. First, we collected concurrent eye movement data on all but one of our seven patients using a 1000 Hz EyeLink eye tracker (SR Research Limited) positioned at the base of the MRI bore. Four patients underwent successful calibration, with accurate data throughout functional MRI runs. For those patients, only 2/5/0/1 eye movements were recorded, defined as a movement of 2° or more towards the scotoma. This accounted for <0.6% of the scan duration i.e. <0.3% of the blind conditions, making any contribution likely to be negligible. To confirm this, when scanner volumes corresponding to eye movements were regressed out of analyses, the results remained unchanged. For the other two patients, we had difficulty with calibration due to their dense field loss. In this situation, direct visualization of the pupil was used via video recording to observe any overt eye movements during the experiment.

Second, all participants performed >90% on a concurrent behavioural task requiring fixation throughout the experiment. Brief colour changes of the fixation cross (300 ms duration) occurred at frequent and random intervals, and participants were given a window of 1 s to press a button connected to the stimulus computer via a parallel port, being specifically instructed not to miss any red crosses or move their eyes around the screen. In addition, before the functional MRI scan all participants took part in behavioural testing lasting at least 60 min, focused on their damaged region of vision. Participants became experienced at maintaining fixation during this assessment. Anyone making even a small eye movement into their damaged hemifield was given specific training not to do so, and it was explained that their data could not be used if this was the case.

Finally, our results show a difference in functional MRI patterns in patients compared to healthy controls and their own intact hemisphere. If eye movements were made, one would expect to see a response in V5/MT+ that matched that for the sighted hemifield, which was not the case. This suggests that eye position cannot account for our findings.

## Results

### Motion in the blind hemifield elicits significant contralateral cortical activation

To quantify responses to visual motion, activation (averaged across coherence levels) relative to baseline fixation was measured for patients and controls. Control participants showed extensive activation throughout the visual cortices (not shown). This pattern was similar to stimulation of the sighted hemifield of patients (Fig. 2D), with activity in V1 and V5/MT+ contralateral to the stimulus, in addition to ipsilateral V5/MT+ activity. When the blind field was stimulated, no discernable activity was seen in early visual cortices. There was, however, significant activation of contralateral V5/MT+ (Fig. 2D), albeit to a considerably reduced spatial extent and percentage signal change compared to the sighted hemifield. The absence of significant activation in ipsilateral V5/MT+ or ipsilateral V1, suggests few, if any, eye movements towards the stimuli.

### Contralateral V5/MT+ shows an unusual pattern of activity during blind hemifield stimulation

Because V1 and V5/MT+ exhibit different patterns of response to motion coherence (Braddick *et al.*, 2001; Handel *et al.*, 2007; Costagli *et al.*, 2014), the percentage BOLD signal change was extracted at each coherence level within regions of interest corresponding to these two visual areas.

**Figure 3:**
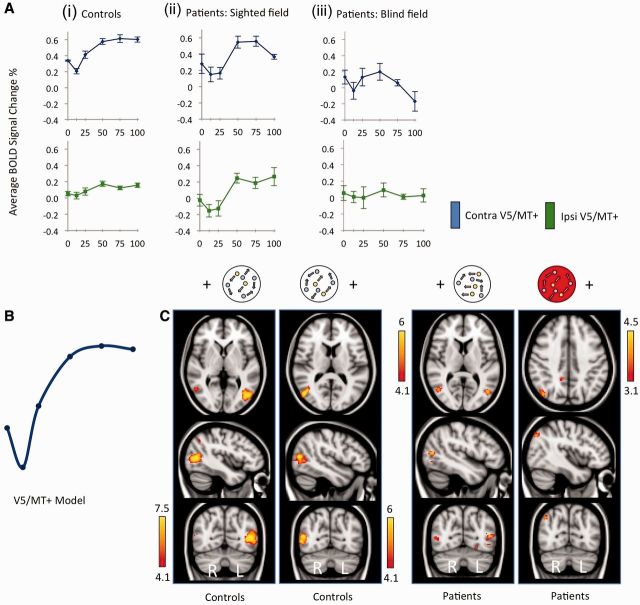
**V5/MT+ response to motion coherence in patients and controls**. (**A**) Graphs show average % BOLD signal change in anatomically-defined V5/MT+ regions of interest as a function of coherence for controls (i), patients’ sighted hemifield (ii), and patients’ blind hemifield (iii). Blue lines represent contralateral V5/MT+ signal change, green represent ipsilateral. Error bars display normalized standard error of mean. (**B**) Schematic depiction of the demeaned data-driven V5/MT+ model for functional MRI response to motion coherence. (**C**) Thresholded activation maps in controls and patients, highlighting regions with a significant relationship with coherence according to the V5 model in **B**. Results for each hemifield are shown separately. The red aperture signifies results for the blind hemifield. Fixed-effects analysis, corrected cluster threshold *P* < 0.05, pre-threshold masking applied to V1 and extrastriate cortex.

In control subjects and in the sighted hemifield of patients, activity in contralateral V5/MT+ showed a significant positive relationship with motion coherence, r = 0.44 (controls, *P* < 0.001), and r = 0.33 (patients, *P* = 0.03). This was as expected from previous research into motion coherence (Rees *et al.*, 2000). However the relationship was not strictly linear, and followed a sigmoid-pattern that was consistent across participants, with strong correlation between the two groups (r = 0.91, *P* = 0.01). Figure 3A (upper row) shows the pattern of response in V5/MT+ in controls and patients to contralateral stimulation.

When the blind hemifield of patients was stimulated, activity in contralateral V5/MT+ no longer showed a positive relationship with increasing coherence. Instead there was a non-significant negative trend (r = −0.22, *P* = 0.16, Fig. 3A). The slope of this fitted regression line differed significantly from that fit to the (positive) pattern in the sighted field (z = 2.5, *P* = 0.01). This was fairly consistent across participants, and showed no correlation with ‘normal’ V5/MT+ activity patterns in controls (r = −0.02, *P* = 0.95) or patients own sighted hemifield (r = −0.13, *P* = 0.4).

### Ipsilateral V5/MT+ response for the sighted hemifield is intact despite V1 damage in the same hemisphere

Some V5/MT+ neurons, particularly those in the medial superior temporal area (Huk *et al.*, 2002) have large receptive fields that cross the vertical meridian into ipsilateral visual space. Indeed, some studies on patients with V1 damage have indicated enhanced ipsilateral representation in the damaged hemisphere (Nelles *et al.*, 2002, 2007). Determining the response of V5/MT+ in the damaged hemisphere raises an important question about whether the altered pattern to motion coherence is an inherent property of that area. An ipsilateral response for the sighted hemifield is likely to be processed by the intact V1, and propagated across the corpus callosum to the damaged hemisphere. The input would, therefore, receive processing beyond that in subcortical regions.

In controls, where response to coherence in the ipsilateral hemisphere has not been systematically investigated in the past, we found a positive sigmoid relationship in V5/MT+, similar to that seen on the contralateral side r = 0.23 (*P* = 0.048, Fig. 3A lower row). As expected, the amplitude of signal was generally lower than contralateral responses. Furthermore, the peak signal in left V5/MT+ was slightly anterior to that with contralateral stimulation (ipsilateral: −44, −70, 6 versus contralateral: −40, −74, 6), which may be indicative of the slightly anterior location of human medial superior temporal relative to MT (Smith *et al.*, 2006).

The same positive sigmoid pattern was also present in the sighted field of patients, where ipsilateral V5/MT+ activity (which was still intact) appeared unaffected by the presence of damage to V1 within the same hemisphere (r = 0.53, *P* < 0.001, Fig. 3A lower row), and correlated highly with ipsilateral activity in the control group (r = 0.93, *P* = 0.007). Notably, this positive linear component was significantly different from the negative trend fit to the same region of interest during stimulation of the blind hemifield (z = 3.4, *P* < 0.001). This implies that it is the input to V5/MT+ that is critical in driving its response pattern. In this case, input most likely comes from the healthy hemisphere, perhaps through interhemispheric callosal connections (which likely involves healthy V1).

### Ipsilateral V5/MT+ response for the blind hemifield seems abnormal

For the blind hemifield of patients, we were interested in whether activity in ipsilateral V5/MT+ more closely resembled healthy positive sigmoid patterns, or the negative trend seen in contralateral V5/MT+. This would allow us to infer specifically whether V1 in the opposite hemisphere was involved in driving ipsilateral V5/MT+ response patterns. In fact, ipsilateral V5/MT+ activity remained very close to baseline across all coherence levels (Fig. 3A), and showed neither a positive nor negative trend with increasing coherence (r = −0.005). The lack of any significant response may indicate that (intact) contralateral V1 is critical for ipsilateral V5/MT+ patterns, however, this cannot be confidently stated based on the current data.

### Parametric analysis using a data-driven V5 model does not fit V5/MT+ activity in the blind hemisphere

The normalized mean signal change within V5/MT+ of control participants was used to generate a data-driven model summarizing the relationship between activation and coherence in contralateral V5/MT+ of the healthy brain, illustrated in Fig. 3B. As the model was generated from V5/MT+, a significant response in this area must follow. However this method allows us to both quantify the specificity of this response pattern, and test for similar patterns in independent data from patients. The model was used as the basis for a parametric general linear model analysis in both groups, carried out separately for each stimulated hemifield. Individual contrasts of parameter estimates for each participant (for example 100% coherence versus baseline) were entered, with explanatory variables weighted according to the model being tested. Additional explanatory variables representing the average response for each participant across conditions were included as regressors of no interest.

Figure 3C indicates the regions showing significant activation that follows the sigmoidal shape. The left panel shows the response in control participants who have significant activity in contralateral V5/MT+ (and ipsilateral in the case of right hemifield stimulation, see Table 2 for details). In addition, significant activity was identified in medial and lateral regions of the frontal pole (Table 2). The right panel indicates significant regions of activity in patients. When the stimulus is in the sighted field, activation in bilateral V5/MT+ fit well with the control model (*P* < 0.0001). However, for the blind ‘left’ hemifield, no activity corresponding to this model was seen in V5/MT+ or early visual cortex. This remained the case even when a more liberal statistical threshold of z = 2.3 was used. Instead, two small clusters were identified in right inferior parietal lobule and right posterior cingulate gyrus (Fig. 3C and Table 2). This suggests that the response pattern to changing coherence in V5/MT+ of patients has a different pattern to healthy V5/MT+. When V1 is damaged, the input underlying responses in this area may come directly from subcortical regions (Sincich *et al.*, 2004). We therefore also compared the response pattern in patients to V1 responses in control participants, where substantial input comes direct from the thalamus.

**Table 2:** MNI coordinates and *z*-statistics for brain areas demonstrating a significant relationship with motion coherence, according to the V5/MT+ or V1 models

	*x*	*y*	*z*	*z*-statistic		*x*	*y*	*z*	*z*-statistic
**Controls**									
**V5/MT+ Model: Left hemifield**					**V5/MT+ Model: Right hemifield**				
Right V5/MT+	50	−70	12	6.67	Left V5/MT+	−42	−68	6	10.31
Left frontal pole	−50	38	10	4.19	Right V5/MT+	54	−60	2	5.32
					Left frontal pole	−4	58	18	4.69
**V1 Model: Left hemifield**					**V1 Model: Right hemifield**				
Right V1	6	−92	2	4.51	Left V1	−18	−66	6	4.01
Right inferior parietal lobule	50	−66	30	4.21	Left insula	−36	−22	−2	4.10
					Right lingual gyrus	14	−54	0	4.20
**Patients**									
**V5/MT+ Model: Blind left hemifield**					**V5/MT+ Model: Sighted right hemifield**				
Right inferior parietal lobule	42	−72	40	4.16	Left V5/MT+	−46	−70	8	5.80
Right posterior cingulate gyrus	12	−46	36	3.79	Right V5/MT+	50	−64	6	5.63
**V1 Model: Blind left hemifield**					**V1 Model: Sighted right hemifield**				
Right V5/MT+	54	−68	12	3.82	Left inferior parietal lobule	−42	−60	42	3.66
					Left precuneus	−10	−58	40	3.57

Only the most significant peak within each area of activation is reported. A statistical threshold of *P* < 0.001, uncorrected for multiple comparisons, was used within V1 and extrastriate cortex (cluster extent threshold >10 voxels). Elsewhere correction for multiple comparisons was made with a cluster threshold of *P* < 0.01, fixed-effects analysis.

### Healthy V1 shows a characteristic response to coherence that is highly correlated to contralateral V5/MT+ in patients

**Figure 4:**
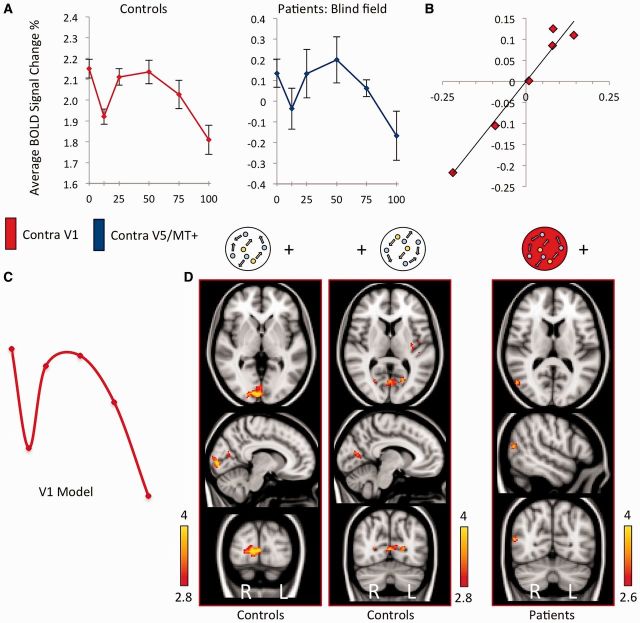
**V1 response to motion coherence in controls, and the cortical regions active according to this V1 model in patients and controls.** (**A**) Average % BOLD signal change as a function of stimulus coherence. The *left* graph (red) shows the response pattern in contralateral V1 of control subjects, averaged across hemispheres. The *right* graph (blue) shows the activation in V5/MT+ of the blind hemisphere in patients. Error bars represent normalized standard error of the mean. (**B**) Correlation of normalized signal change in contralateral V5/MT+ for patients blind hemifield, versus contralateral V1 activation in healthy controls, r = 0.98, *P* < 0.01. (**C**) Schematic of demeaned data-driven V1 model from control subjects for functional MRI response to motion coherence. (**D**) Active brain regions showing a significant relationship with motion coherence according to the V1 model in **C**. Results for each hemifield are shown separately for controls, and patients blind hemifield (highlighted by the red aperture). Fixed-effects analysis, corrected cluster threshold *P* < 0.05, pre-threshold masking applied to V1 and extrastriate cortex.

The motion stimulus significantly activated contralateral V1 in controls and in the sighted hemisphere of patients. Response magnitude as a function of coherence in control participants, quantified by per cent change in BOLD signal, is shown in Fig. 4A. The left graph indicates a weak negative correlation overall (r = −0.12), with a significant decrease in signal change in the coherent condition compared to random motion (paired *t* = 3.4, *P* = 0.006). This relationship again appeared non-linear, with a nadir in activity for coherence levels of 12.5%.

In patients, this pattern of negative association with coherence seen in control V1 was also present in V5/MT+ on the side without functioning V1. There was a strong and significant correlation between control participant V1 activity and the pattern of activity in V5/MT+ on the lesion side during blind hemifield stimulation (r = 0.98, *P* < 0.001). This relationship is shown in Fig. 4B and is in stark contrast to the absence of any correlation with normal V5/MT+ patterns.

### Parametric analysis using a data-driven V1 model fits V5/MT+ activity in the blind hemisphere

Using an analogous method to that illustrated in Fig. 3, a normalized V1-data driven model was generated from contralateral V1 responses in control participants, and applied to both control and patient groups (Fig. 4C). This V1 model shows an initial dip similar to the V5/MT+ model, but then shows a considerable decrease with higher coherence levels. In control participants no regions of extrastriate cortex showed activity conforming to the V1 model. However, there was such activation in the left insula, which has previously been shown to exhibit a negative linear relationship with motion coherence (Rees *et al.*, 2000), and right inferior parietal lobule (Table 2).

When the V1 model was applied to the blind field of patients, the only occipital area conforming to this pattern was contralateral cortex corresponding to V5/MT+ (z = 3.82, *P* < 0.001 uncorrected. See Fig. 4C, and Table 2 for coordinates). No other region showed a significant association with this model. This finding remained significant when analysed using a mixed effect model (z = 3.52, *P* < 0.001), implying the result to be robust across the patient group.

### A weighted-linear model can accurately predict V5/MT+ response

Finally, we had also predicted that weaker non-striate projections to V5/MT+ may contribute to average neuronal responses, but are likely to be overshadowed by the much greater input from V1. We have now revealed that when V1 is absent due to damage in adulthood, V5/MT+ responds in a pattern typical of (normal) early visual cortex. According to our prediction, this pattern of activity should also contribute to average V5/MT+ responses in healthy control subjects, who also possess a direct subcortical connection, but whose activity is normally dominated by input from V1. Indeed, we are able to generate a highly predictive model of control V5/MT+ BOLD response patterns by simply taking a linear combination of: (i) a direct linear relationship between signal change and coherence, as predicted from studies measuring neuronal electrophysiology responses in MT; and (ii) the average response pattern in patient V5/MT+ when V1 is damaged (postulated to reflect direct, residual subcortical input).

**Figure 5:**
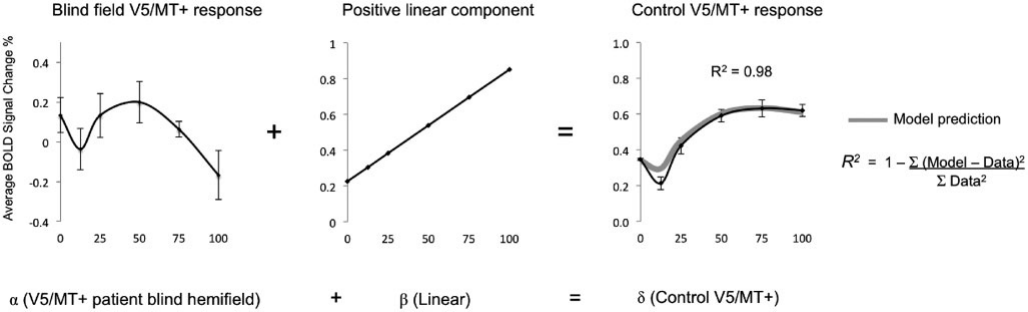
**Activity in V5/MT+ can be accurately predicted from a weighted-linear model**. V5/MT+ activity in patients during blind hemifield stimulation can be combined with a simple positive linear component to predict BOLD signal change in V5/MT+ of controls to a high degree of precision. Relative weightings are signified here by α, β, and δ symbols.

Using this model, we are able to account for 98% of the variance in control V5/MT+ functional MRI signal change (Fig. 5). This equation can also be rearranged to model the abnormal V5/MT+ responses in patients, defined by a loss of the positive linear component in control V5/MT+ responses, which also accurately predicts the data (R^2 ^= 0.93), or indeed the positive linear component itself (R^2 ^= 0.98).

BOLD signal is an indirect measure of neural activity that reflects a population average and is likely to combine signals even at the level of a single voxel. Separately driven signals within the same region of interest may be embedded in average BOLD responses, but may only be revealed if the individual signal components are fully understood.

## Discussion

Here we demonstrated that area V5/MT+ has a different response pattern to motion coherence when V1 is damaged and can no longer modulate input to this area. The similarity to response patterns in V1 measured in control participants suggests that it may be direct subcortical input that shapes the motion coherence response in patients under these conditions.

It is already recognized that certain aspects of motion processing in V5/MT+ are retained in the absence of V1. Neurophysiological studies in non-human primates show that a considerable proportion of direction-selective neurons retain their responsiveness following striate cortex removal or cooling, even though strength and selectivity of responses is decreased (Rodman *et al.*, 1989; Girard *et al.*, 1992). Thus, V5/MT either has its own intrinsic circuitry sufficient to produce directionally selective responses from non-selective input, or, more likely, it can rely upon direction-selective input from regions other than V1, such as superior colliculus and pulvinar (Rodman *et al.*, 1986; Kato *et al.*, 2011). The organization of such non-striate pathways, however, remains largely unknown. Whether they can support more complex V5/MT properties such as global motion processing remains to be determined.

### The majority of studies suggest a linear relationship between motion coherence and V5/MT+ response

In macaque V5/MT, many direction-selective neurons show an approximately linear relationship between moving visual stimuli with increasing motion coherence and firing rate (Britten *et al.*, 1993). Of the remaining neurons, roughly equal proportions demonstrate either negatively or positively accelerating non-linearities, of variable strength. In human studies, PET, magnetoencephalography and functional MRI have offered a more indirect and global measure of V5/MT+ neuronal populations, and have largely concurred that activity increases with coherence (Cheng *et al.*, 1995; Rees *et al.*, 2000; Braddick *et al.*, 2001; Nakamura *et al.*, 2003; Aspell *et al.*, 2005; Handel *et al.*, 2007; Becker *et al.*, 2008). Given the range of neuronal subtypes and experimental protocols, consistent findings of a linear relationship are perhaps surprising. Indeed, on closer inspection some studies have found a dip in activation at low coherence levels similar to that seen in the current data (Handel *et al.*, 2007; Siegel *et al.*, 2007), which may be missed if fitted to a purely linear model. In this study, we have shown that functional MRI activity in V5/MT+ may reflect an average of different response patterns, one of which may represent a direct linear relationship with coherence.

There are also several studies reporting a negative relationship with coherence in human V5/MT+ (McKeefry *et al.*, 1997; Previc *et al.*, 2000; Harrison *et al.*, 2007), or no difference between coherence and noise (Smith *et al.*, 2006). Two factors in particular may be important in defining such a relationship: stimulus size and density. A small 4–8° diameter aperture is typically associated with a positive relationship (Rees *et al.*, 2000; Becker *et al.*, 2008) whereas stimuli subtending a wider visual field (>20°) have generated the opposite pattern (McKeefry *et al.*, 1997; Previc *et al.*, 2000; Harrison *et al.*, 2007). This may occur because some direction-selective neurons in V5/MT exhibit surround inhibition (Tanaka *et al.*, 1986; Raiguel *et al.*, 1995). Therefore a wide field containing coherent motion may stimulate directionally-selective inhibitory surrounds, whilst smaller apertures fall solely within the ‘classical receptive field’, eliciting no suppressive signals (Tanaka *et al.*, 1986). However, this explanation alone can neither account for all negative studies, nor those reporting a positive relationship using large stimuli (Cheng *et al.*, 1995; Nakamura *et al.*, 2003; Aspell *et al.*, 2005; Becker *et al.*, 2008).

Similarly, in the majority of cases, sparse stimuli ≤1 dot/°^2^ result in a negative response in V5/MT+ (McKeefry *et al.*, 1997; Previc *et al.*, 2000; Smith *et al.*, 2006; Harrison *et al.*, 2007). A weak switch in direction was also demonstrated in one study comparing fairly sparse (1 dot/°^2^) to dense (6 dot/°^2^) parameters (Becker *et al.*, 2008). Two exceptions used comparable 60° sparse displays and found greater activity with coherence in regions corresponding to V5/MT+ (Cheng *et al.*, 1995; Nakamura *et al.*, 2003), restricted in one study to the right hemisphere (Cheng *et al.*, 1995). It is possible that in these two cases, the large stimuli led to greater activation in the medial superior temporal area, known to possess much wider receptive fields (Tanaka *et al.*, 1986) and respond particularly strongly to patterns of global motion such as rotation and expansion (Heuer and Britten, 2007).

In the macaque, average receptive field size in V5/MT ranges from 0.6–8° diameter at equivalent eccentricities to the stimuli used here (up to 12°; Albright and Desimone, 1987). Therefore if one considers a similar sized sparse display such as Harrison *et al.* (2007) with dots positioned an average of 5° apart, both V1 neurons and most of those in V5/MT+ would be stimulated by just one dot per receptive field. It may therefore not be surprising that with such a sparse display, V5/MT+ responds in the same way as V1 even in the presence of 100% coherence.

### Healthy V1 shows a distinct characteristic response to motion coherence

We identified a weak negative trend with coherence in V1 of healthy participants, which was strongly correlated with the pattern of coherence-related activation in V5/MT+ contralateral to the blind visual field in patients. Whilst V1 has not been investigated as extensively as V5/MT+ in the past, similar negative responses have been reported in several human imaging studies (Braddick *et al.*, 2001; Handel *et al.*, 2007; Harrison *et al.*, 2007; Costagli *et al.*, 2014). Neurophysiological research has not examined this specifically, but V1 direction selective cells fire when a preferred direction component is present in the receptive field and do not show significant suppression if opposing motion is also present (Snowden *et al.*, 1991). It has also been suggested that surrounding V1 neurons can show an enhanced response in the presence of opposing motion, due to the presence of increased contrast energy (Heeger *et al.*, 1999). Thus if a single neuron fires similarly with coherent or incoherent motion, it may be inferred that the negative pattern in functional MRI reflects a population average, perhaps influenced by the range of directions of motion present within any given voxel and the proportion of directionally selective cells that are activated over time.

In the current experiment non-coherent stimuli had completely random trajectories of motion. Conversely, coherent stimuli moved in eight directions (45° apart), lasting 2 s each. It is possible that over time, and in regions that do not exhibit significant summation and/or subtractive inhibition, a greater proportion of directionally-selective neurons were activated by the greater range of directions during incoherent conditions. This situation would be similar to Braddick *et al.* (2001) and Handel *et al.* (2007) which both compared coherent motion in two directions to random noise, and even Harrison *et al.* (2007) who used two directions in the coherent condition, compared to four in the incoherent condition. An alternative explanation is that top–down signals from higher visual areas cause suppression, perhaps due to prediction error (Handel *et al.*, 2007; Harrison *et al.*, 2007). This could still account for the results in patients if, for example, back-projections arise from the posterior cingulate gyrus where significant activity was seen in our V5/MT+ model (Table 2), as in Harrison *et al.* (2007). The current data, however, do not allow these possibilities to be distinguished.

### Differences between neural responses to motion in V1 and V5/MT+

Both neurophysiological and human neuroimaging studies show that motion-related responses of V5/MT+ differ qualitatively from those in V1 in several respects. In particular, responses in V5/MT+ appear to correspond more closely to visual perception than those in V1 (Shadlen and Carney, 1986). One example particularly relevant to the current data is that neurons in the two areas typically respond differently to global and component features of motion. In the rhesus monkey, ∼30% of neurons in V1 are direction-selective and respond largely to motion of the ‘components’ within complex patterns rather than the pattern as a whole (Snowden *et al.*, 1991, 1992; Movshon and Newsome, 1996). This has led to the suggestion that these cells act as ‘local motion energy filters’ or even a ‘gateway’ responding to particular bandpass limits for orientation, spatial and temporal frequency, serving to minimize noise from incoherent motion in V5/MT+ (Handel *et al.*, 2007). In contrast, ‘global’ motion computation seems to rely on combining of local component measures across space and time (Reppas *et al.*, 1997), and has been suggested to occur after V1, perhaps within MT itself (Movshon and Newsome, 1996; Huk and Heeger, 2002).

How these ‘global’ receptive fields in V5/MT+ are informed by V1 signals is described via a motion opponency or linear–non-linear cascade model in the macaque (Simoncelli and Heeger, 1998; Rust *et al.*, 2006; Tsui and Pack, 2011). However, further work is needed to translate this to the human visual system and the interpretation of functional MRI BOLD signal change. Here we have shown that V1 is essential to generate a positive relationship with coherence in contralateral V5/MT+. Identical stimulus parameters presented in patients and controls elicited very different patterns of activity, whose fundamental difference was the presence of a positive linear component (Fig. 5). Furthermore, the same ipsilesional V5/MT+ voxels in patients responded in opposing directions dependent upon whether the blind or sighted visual field was stimulated. This implies that a normal response to global motion in V5/MT+, unlike direction-selectivity, does seem to depend upon contralateral V1 and cannot be derived through direct subcortical pathways. Instead, the relative receptive field size and a hierarchical model of converging inputs can be used to explain the response to global motion in these results and the majority of studies described. This accounts for the positive linear relationship with coherence; however, we have also shown that non-global components may contribute to average V5/MT+ responses. These may reflect direct subcortically driven responses, and/or a proportion of neurons that lack summation of V1 signals.

The simplest explanation of the role of V1 presented here is that it is the loss of V1 ‘input’ to V5/MT+ that alters the pattern of activity to motion coherence. However, we cannot exclude the possibility that the critical visual area for the motion coherence response is not V1, but is perhaps modulated by V1. The lack of other extrastriate visual areas that exhibit patterns of response consistent with either V1 or V5/MT+ (Figs 3 and 2, respectively) makes this interpretation even less likely.

There is an alternate theory that feedback from areas such as V5/MT+ to V1 is required for processing local details, which cannot be computed by the large receptive fields of extrastriate neurons (Bullier, 2001; Hochstein and Ahissar, 2002). Those models suggest that awareness of global motion features would precede awareness of local detail, computed in V1. Our findings are not entirely consistent with this theory, since in the absence of V1, normal global processing in V5/MT+ is no longer possible. Rather, the current data are more consistent with two transcranial magnetic stimulation studies suggesting two critical periods of V1 activity; one preceding and one postdating V5/MT+ activity (Silvanto *et al.*, 2005*a*, *b*). In this scenario, V1 may be required for the initial computation of local details, before feeding forward to V5/MT+ for global motion processing. Subsequent feedback to V1 may then induce a conscious experience. Although it is not possible to uncover the latter point from the current study, reciprocal connections may be important for conscious perception, impaired in patients with V1 damage. Blindsight occurs largely without awareness, and may be more akin to the intermediate reports of participants in the transcranial magnetic stimulation study, when V1 was stimulated before V5. In this condition, even though phosphenes remained largely stationary, participants were not always confident that they were not moving (Silvanto *et al.*, 2005*a*).

### Subcortical pathways carry motion information directly to V5/MT+

V5/MT+ activation in the presence of (unilateral) V1 damage demands that a mechanism is in place to relay information from corresponding regions of the retina to extrastriate cortex. Numerous non-human primate studies have suggested that direct routes may exist from the lateral geniculate nucleus (Sincich *et al.*, 2004; Schmid *et al.*, 2010) or superior colliculus and pulvinar (Lin *et al.*, 1974; Benevento and Rezak, 1976; Trojanowski and Jacobson, 1976; Maunsell and Van Essen, 1983; Rodman *et al.*, 1986; Kato *et al.*, 2011). Such subcortical pathways could certainly account for our finding of V5/MT+ activation in patients, in particular given that patterns change to resemble early visual cortex of healthy controls. Little is known about the organization of non-striate inputs to V5/MT+. However, subcortical input may follow a similar organization to the geniculate innervation of V1, with a lack of convergence across receptive fields that is likely to be important in the projections between V1 and V5/MT+. It has been postulated that similar alternate pathways may account for the phenomenon of blindsight in humans (Weiskrantz *et al.*, 1974; Cowey, 2010), and techniques such as latency analysis of visual evoked potentials, EEG and functional MRI signals have been used to infer such connectivity (ffytche *et al.*, 1995, 1996; Gaglianese *et al.*, 2012) as well as MRI diffusion-based tractography (Bridge *et al.*, 2008, 2010). All of the patients in this study sustained cortical damage during adulthood, when one may expect that the potential for plasticity is considerably reduced. Furthermore, approximately half of cases underwent testing within 1 year of lesion onset. When considered in light of the breadth of evidence for a direct subcortical connection to V5/MT+, we suggest this is most likely to represent intact residual pathways in the healthy visual system, rather than a change in connectivity.

In conclusion we have shown that in the absence of V1, V5/MT+ response to global motion coherence resembles patterns in primary visual cortex, perhaps shaped by similar direct subcortical input. We have also confirmed predictions from animal studies that V1 is essential for typical V5/MT+ global motion responses. It is likely that a direct subcortical pathway to V5/MT+ does exist in the intact visual system, but may normally be overshadowed by the overwhelming input from V1. By studying patients with V1 damage, we have been able to reveal their influence, which may be useful in developing more accurate models of visual cortex responses.

## Abbreviations

BOLD: blood oxygen level-dependent
MT: middle temporal
V1: primary visual cortex
